# Advancements and Challenges in IoT Simulators: A Comprehensive Review

**DOI:** 10.3390/s24051511

**Published:** 2024-02-26

**Authors:** Reham Almutairi, Giacomo Bergami, Graham Morgan

**Affiliations:** 1 Faculty of Science, Agriculture and Engineering, School of Computing, Newcastle University, Newcastle upon Tyne NE4 5TG, UK; giacomo.bergami@newcastle.ac.uk (G.B.); graham.morgan@newcastle.ac.uk (G.M.); 2 College of Computer Science and Engineering, University of Hafr Al Batin, Hafr Al Batin 31991, Saudi Arabia

**Keywords:** IoT simulators, IoT simulation challenges, IoT simulators performance

## Abstract

The Internet of Things (IoT) has emerged as an important concept, bridging the physical and digital worlds through interconnected devices. Although the idea of interconnected devices predates the term “Internet of Things”, which was coined in 1999 by Kevin Ashton, the vision of a seamlessly integrated world of devices has been accelerated by advancements in wireless technologies, cost-effective computing, and the ubiquity of mobile devices. This study aims to provide an in-depth review of existing and emerging IoT simulators focusing on their capabilities and real-world applications, and discuss the current challenges and future trends in the IoT simulation area. Despite substantial research in the IoT simulation domain, many studies have a narrow focus, leaving a gap in comprehensive reviews that consider broader IoT development metrics, such as device mobility, energy models, Software-Defined Networking (SDN), and scalability. Notably, there is a lack of literature examining IoT simulators’ capabilities in supporting renewable energy sources and their integration with Vehicular Ad-hoc Network (VANET) simulations. Our review seeks to address this gap, evaluating the ability of IoT simulators to simulate complex, large-scale IoT scenarios and meet specific developmental requirements, as well as examining the current challenges and future trends in the field of IoT simulation. Our systematic analysis has identified several significant gaps in the current literature. A primary concern is the lack of a generic simulator capable of effectively simulating various scenarios across different domains within the IoT environment. As a result, a comprehensive and versatile simulator is required to simulate the diverse scenarios occurring in IoT applications. Additionally, there is a notable gap in simulators that address specific security concerns, particularly battery depletion attacks, which are increasingly relevant in IoT systems. Furthermore, there is a need for further investigation and study regarding the integration of IoT simulators with traffic simulation for VANET environments. In addition, it is noteworthy that renewable energy sources are underrepresented in IoT simulations, despite an increasing global emphasis on environmental sustainability. As a result of these identified gaps, it is imperative to develop more advanced and adaptable IoT simulation tools that are designed to meet the multifaceted challenges and opportunities of the IoT domain.

## 1. Introduction

In the past two decades, the Internet of Things has rapidly evolved, promising to transform the way we live, work, and interact with our environment. As a whole, the Internet of Things refers to a network of physical objects that are connected and exchange data via the internet with other devices and systems through the use of sensors, software, and other technologies. Items such as household items and industrial tools can be included in this category. The overarching goal of IoT is to create a smarter, more efficient world by seamlessly integrating the physical and digital universes. The idea of interconnected devices is not new. However, the term “Internet of Things” was coined in 1999 by Kevin Ashton, co-founder of the Auto-ID Center at MIT [1]. Ashton’s vision was rooted in the belief that when objects can sense the environment and communicate, they could bring about unprecedented efficiency and automation. This vision was propelled by the rapid advancements in wireless technologies, decreasing computing costs, and the proliferation of mobile devices. Today, with billions of devices connected globally, his vision is increasingly becoming a reality, reshaping industries and daily life.

### Contributions

The objectives of the current study are not only to find out about existing and updated IoT simulators, analyze them, and study their uses in real-case scenarios but also to evaluate the most recent open-source IoT simulators based on specific criteria that play a crucial role in IoT development. This review emphasizes open-source simulators to ensure the accessibility of analysis and customization options for researchers and practitioners. The IoT simulation field has been subjected to substantial research; however, most of these studies focus on specific aspects of the field such as the healthcare domain, security aspects, or wireless sensor techniques. Few comprehensive reviews have been published that examine IoT simulators in a broader context, considering performance evaluation metrics critical for IoT development, such as device mobility, energy models, Software-Defined Networking (SDN), heterogeneity, and scalability. To the best of our knowledge, no review paper examines the ability of IoT simulators to support renewable energy sources or use them in the VANET simulation field. So, researchers need an updated review that evaluates the current IoT simulators based on recent technological developments. This review is a valuable resource for researchers and practitioners seeking to understand the current landscape of IoT simulation tools. So, our objective in this review is to evaluate IoT simulators’ ability to simulate complex and large-scale IoT scenarios and to ensure that they meet the specific requirements, including support mobility, energy model, SDN, scalability, and renewable energy sources. We also aim to identify the open challenges in IoT simulators that still need to be addressed.

The results of our literature review indicate that the IoT simulator landscape has seen a diverse range of tools, each with its capabilities and features, indicating that an appropriate generic IoT simulator that can simulate any scenario across different IoT applications is lacking. Also, there are still several challenges to overcome. These challenges include security and privacy issues, the need for supporting mobility, SDN, and energy models, and addressing scalability and heterogeneity. Moreover, with the recent evolution of cities and traffic, more focus should be placed on integrating IoT simulations with VANET simulations. Currently, only one study examined the integration of the IoT simulator *IoTSim-OsmosisRES* [2] with traffic simulation to simulate VANET environments. Given these challenges, there’s an urgent demand for tools that can adapt to the continuously evolving requirements of the Internet of Things. Addressing these issues will enable more comprehensive and accurate simulations and open a variety of opportunities for researchers to make significant contributions to the field. The future development of IoT simulators is likely to be dynamic and multifaceted, reflecting the complexities and opportunities of the rapidly advancing IoT landscape.

From our analysis, it is evident that there is no generic IoT simulator that is able to simulate any scenario in different IoT applications. Moreover, mobility, a crucial aspect of IoT, is supported by only a few simulators, with some offering only partial modeling of mobile IoT environments. The adoption of Software-Defined Networking (SDN) is still in its early stages, with only a few simulators offering comprehensive support. Energy modeling, a critical component for sustainable IoT deployments, varies in depth and focus across simulators. While some simulators provide detailed energy modeling for specific environments, others have a broader approach. In particular, *IoTSim-OsmosisRES* stands out as the first IoT simulator supporting renewable energy sources (RES), followed by *SimulatorBridger*; these are the only two simulators that can support RES and offer a comprehensive framework for evaluating Autonomic Computing algorithms in the context of osmotic computing. Scalability remains challenging for some simulators, but tools like the NB-IoT simulator are explicitly designed for large-scale environments, showcasing the potential for simulating expansive IoT networks.

The remainder of this paper is organized as follows: Section 2 provides some background information on the IoT architecture and attributes. Next, Section 3 details the importance of simulations in IoT environments and their associated challenges. Then, Section 4 explains the research protocol used in this survey, and Section 5.1 presents a summary of the studied literature and reviews the most up-to-date IoT simulators. After that, the following two sections highlight the answers to some research questions by identifying the categories of IoT simulators Section 5.2 and exploring the main features of simulators in different IoT applications Section 6. Based on specific evaluation criteria, we perform a deep comparison of the existing IoT simulators Section 7. Finally, Section 8 discusses current challenges and future works in IoT simulation.

## 2. IoT Fundamentals

### 2.1. IoT Architecture

**Figure 1:**
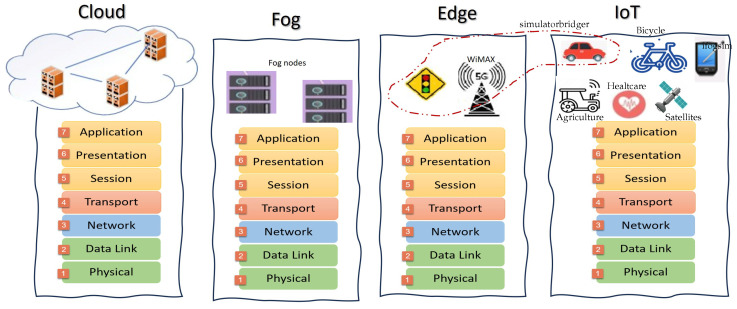
Network and IoT components [4,5,6,7,8,9].

In this section, we provide a brief overview of the IoT framework. It is important to note that not all applications or technologies employ a standard IoT architecture. There is a unique framework for each technology and each claims to be the best. IEEE proposed a draft of the IoT architecture framework for smart cities and a smart grid architecture standard from 2018 to 2019. Navigating the complexity and expansive nature of the Internet of Things (IoT) presents a challenge due to its inherent heterogeneity and extensive scalability. In constructing an IoT ecosystem, it is crucial to integrate a variety of elements, including devices, networks, and applications, to ensure they work in harmony to facilitate intelligent outcomes, while also prioritizing the aspect of security. IoT communication is underpinned by several layers involving a mix of technologies, protocols, and standards. These layers enable disparate technologies to interact across numerous scenarios, fostering scalability, diversity, and seamless interoperability within IoT systems as noted in the literature [3]. The architecture of the IoT typically consists of four essential components: the service, platform, network, and device layers, with many research institutions adopting this standardized IoT framework as depicted in Figure 1 to maintain consistency and expertise in IoT development.

The service layer, positioned at the forefront, is designed to enhance user interaction through an interface, facilitating communication with users. This layer includes a variety of services such as autonomous driving, healthcare, smart industries, personal devices, and door security systems, all connected to a platform layer that delivers services customized for the user. Following the service layer is the platform layer, which underpins IoT applications and services. This layer is diverse, consisting of different platforms including device, data analysis, service development, and service platforms. For instance, the device platform provides an environment for the execution and development of services for users. Conversely, the data analysis platform supports context understanding and forecasting, enables entity collaboration, and bridges communication between the service layer and other layers by converting natural language into a format understandable by machines. Additionally, the service development platform offers development toolkits to simplify the creation of IoT services, while the service platform facilitates the generation and operation of various applications. Beyond these, the network layer plays a critical role in the IoT framework, responsible for the transmission of data across devices, content, services, and users. It handles the processing, control, and management of extensive network traffic. Finally, the device layer is engaged in sensing environmental conditions through various sensors, processing this data for forwarding to a sink node or gateway, and responding as necessary. The device must embody intelligence by implementing autonomous actuation and a sophisticated control algorithm. The physical layer ought to be able to obtain and manage IoT devices. Beyond the four layers, the significance of security and privacy in IoT cannot be overstated. Rather than designating security as a separate layer, each layer should embed security solutions to shield it from potential threats. Security concerns must be regarded as a crucial functional component for each layer, and any existing or future solutions should be tailored to each layer’s specific characteristics and functions [10]. Every layer is important, possessing distinct roles and capabilities to facilitate IoT. While each IoT layer warrants thorough discussion, this paper will specifically delve into the IoT network. Our primary emphasis will be on exploring the challenges of the IoT network and offering foresight for its future development.

### 2.2. Attributes of the IoT Infrastructure

The Internet of Things (IoT) emerges as a transformative paradigm, orchestrating an intricate web of interconnected devices spanning the spectrum from rudimentary sensors to advanced computational apparatuses throughout an expansive, distributed network. Characterized by its heterogeneous device environment, the IoT seamlessly combines low-power, resource-constrained sensors with robust computational devices, enabling both implicit and explicit interactions among them. This network, which is projected to develop into an ultra-large-scale system, navigates through the multifaceted challenges of managing real-time interactions, ensuring stable connectivity in a dynamic, frequently infrastructure-less network, and handling large amounts of event data. Moreover, the intrinsic characteristics of context awareness, intelligence, and location sensitivity in IoT devices and applications enable the creation of more adaptive, autonomous, and personalized smart environments. This complex infrastructure of the IoT holds the potential to reshape our digital interactions, embedding deeper intelligence and functionality into the internet-connected world that envelops us.

- Diverse Devices: IoT incorporates a wide array of devices, ranging from low-cost, low-power radios, which often avoid using WiFi or conventional cellular networks, to more robust computing devices necessary for tasks like routing and data processing. The diversity in devices arises not only from varying capabilities and features but also from the involvement of multiple manufacturers and distinct application needs [11].
- Limited Resources: The compact form factor of embedded computing and sensors restricts their processing, memory, and communication capabilities. For instance, while RFID devices might lack processing capabilities or even a power source, devices that offer more resources tend to be larger and more costly [12].
- Unplanned Interactions: IoT applications can facilitate unexpected interactions as devices move and enter the communication range of others, spontaneously generating events. An example would be a smartphone user unknowingly triggering events when approaching smart home appliances.
- Extensive Network and Event Volume: The IoT environment can involve thousands of interacting devices within a single locale, such as a building or supermarket, and on a global scale, it represents an ultra-large-scale network with potentially trillions of nodes. The vast number of interactions and events can pose challenges like event congestion and diminished event processing ability [13].
- Dynamic, Infrastructure-less Network: The IoT will amalgamate devices, many of which will be mobile and constrained in resources. The network will be highly dynamic due to factors like mobile nodes, unstable wireless connections, and battery limitations, making it challenging to maintain a stable network for various application scenarios [11].
- Context Sensitivity: Sensitivity to Context: The significance of context cannot be overstated in the realm of IoT applications. The vast quantities of data produced by a multitude of sensors attain their true value only through careful analysis and interpretation. The concept of context-aware computing is essential here; it involves the storage of information pertinent to sensor data, thereby enabling the interpretation that is critical for the adaptive and self-governing behaviors of IoT components [14].
- Intelligence: Smart Capabilities: Reflecting Intel’s vision for the IoT, the foundation of any IoT system is its intelligent devices and systems. Within the fluid and expansive network of the IoT, it is essential for smart entities, along with other components such as Web services and virtual objects, to work together seamlessly and possess the ability to act autonomously, making decisions based on the context and surrounding environment [11].
- Location Consciousness: Location information is crucial in IoT, significantly impacting context-aware computing. In a vast network, interactions are heavily influenced by entities’ locations as well as the one of their surroundings.
- Distributed Nature: The IoT is a globally distributed network like the traditional internet. Its pronounced spatial dimension ensures that the network is distributed on various scales, both globally and locally, within a specific application area.

## 3. IoT Simulations

### 3.1. The Importance of IoT Simulation

As the Internet of Things (IoT) has become an important technology, enabling communication between devices and systems across various sectors, such as healthcare, agriculture, manufacturing, and smart cities, IoT simulators play a crucial role in the development, testing, and research within the IoT domain for several reasons [15,16]:

- Scalability Testing: IoT networks often involve many devices. Simulators allow developers to test how systems perform under the stress of thousands or even millions of connected devices without needing to deploy physical devices. In addition, simulating devices distributed across various geographical locations helps understand and optimize data flow, latency, and management across different regions.
- Cost-Effective Development: Physical devices can be expensive and logistically challenging to manage in a testing environment. Simulators help in reducing costs related to purchasing, setting up, and maintaining physical devices. Also, simulators allow developers to utilize resources more efficiently, as they can test various scenarios and configurations without additional hardware investments.
- Risk Mitigation: Simulators enable developers to introduce faults and observe system responses, which is crucial for creating robust IoT systems without risking actual devices and data. Moreover, simulating cyber-attacks or security breaches on an IoT network helps identify vulnerabilities and enhance security features without exposing devices to threats.
- Prototyping and Validation: Developers can use simulators to create virtual prototypes of IoT systems, enabling them to test and validate functionalities before physical deployment. Also, simulators allow the testing of various use cases and scenarios to validate the feasibility and functionality of IoT solutions in a controlled environment.
- Interoperability Testing: IoT ecosystems often involve diverse devices with varying capabilities. Simulators enable interoperability testing among these devices without needing all of them physically. In addition, different IoT devices may use different communication protocols. Simulators facilitate testing the compatibility and interoperability of these protocols within the IoT network.
- Real-world Scenario Testing: Simulators can mimic different environmental conditions (like temperature, humidity, etc.) to test how devices and systems perform under various scenarios. Additionally, simulating different user behaviors and interactions with IoT devices helps understand usage patterns and optimize user experience.
- Research and Innovation: Researchers can use simulators to experiment with new algorithms, communication protocols, and architectures in a risk-free environment. Also, simulators can generate data that researchers can analyze to derive insights into system performance, user behaviors, and other aspects without deploying a full-scale physical setup.
- Training and Learning: Simulators provide a practical learning environment for students and professionals to understand IoT concepts and technologies without needing physical labs. Moreover, developers, testers, and administrators can enhance their skills by working with simulators to understand the complexities and challenges in IoT ecosystems.

### 3.2. Challenges in IoT Simulation

Simulating the Internet of Things (IoT) brings about several challenges that need careful attention to ensure the simulations are accurate and can handle large-scale operations [17,18,19].

- Security Challenges: The IoT involves sharing sensitive data between various devices and sensors, which can be susceptible to cyber-attacks. Simulations need to account for these security threats and accurately mimic the actions of potential attackers. Understanding various possible attacks and being able to model these in simulations is crucial.
- Energy Consumption Challenges: Many IoT devices rely on battery power, which limits their available energy. It is crucial for simulations to accurately depict the energy consumption of these devices and how their behavior impacts the network’s overall energy usage. Gaining insights into and modeling the energy consumption patterns of various devices and sensors is vital. Additionally, the extensive nature of IoT systems often renders centralized management of process migration and data flow control inefficient. As such, there’s a pressing need for the development of mechanisms that facilitate the use of distributed management algorithms. Moreover, with the unpredictable nature of environmental conditions—such as wind speed and solar radiation levels—simulators must be adaptable to changes in the operational environment of the devices. Modeling the diversity of green energy sources is important, moving beyond treating energy as a pre-existing factor. This includes considering all energy-related metrics, not just focusing on cost and consumption but also on the self-sufficiency provided by renewable energy sources.
- Heterogeneity Challenges: The IoT landscape is characterized by the integration of devices and sensors from various producers, each employing distinct communication standards. Simulations are tasked with navigating these variances, ensuring an accurate representation of every network component’s behavior. A thorough comprehension of the myriad communication protocols within the IoT, alongside the capability to simulate device behaviors governed by these protocols, is indispensable. For instance, in the context of supporting Software-Defined Networking (SDN), the employment of a multi-tier architecture that includes IoT, edge computing, cloud services, and SD-WAN necessitates harmonized interaction across these strata. Consequently, each layer is marked by continuous evolution, characterized by a mix of components, data formats, and protocols. This complexity may extend to varying behaviors and configuration specifics, such as power sources, computational/storage capabilities, and network bandwidth [20].
- Scalability Challenges: The IoT environment is characterized by a vast and ever-growing number of interconnected devices, each generating, processing, and transmitting data. As the number of devices and their interactions increase, the complexity of the simulation grows exponentially. Traditional simulation tools and methodologies designed for smaller and less dynamic networks often struggle to model the intricate behaviors and interactions of large-scale IoT networks accurately. This scalability challenge is further compounded by the heterogeneity of IoT devices, ranging from simple sensors to sophisticated computing devices, each with unique communication protocols, processing capabilities, and power constraints. To effectively simulate such diverse and expansive environments, there is a pressing need for innovative simulation techniques that can accommodate the vastness and variability of the IoT landscape, while ensuring accuracy, efficiency, and real-time responsiveness.

## 4. Review Methodology 

**Figure 2:**
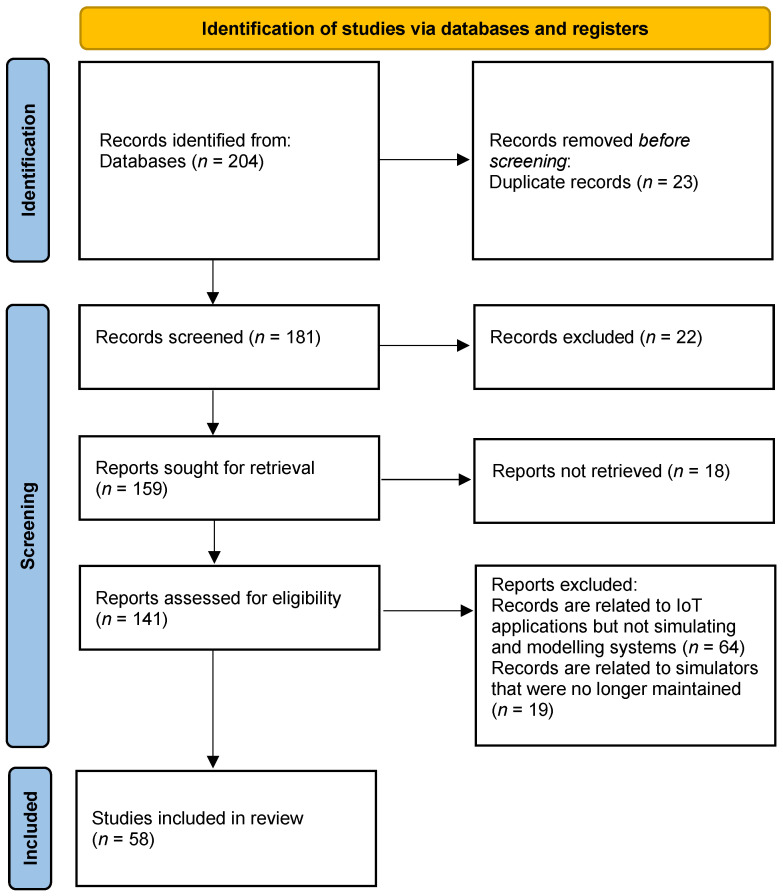
PRISMA flow diagram of current study.

This section outlines the methodology employed in conducting this comprehensive literature review on Internet of Things (IoT) simulators. This research uses the systemic review and meta-analysis by PRISMA standards [21]. A successful PRISMA schematic diagram is generated in Figure 2.

Our literature review aimed to provide a comprehensive and up-to-date overview of the IoT simulator landscape. This involved extensively exploring academic journals, conference proceedings, technical reports, industry publications, and online repositories. The goal was to collect a wide range of scholarly and industry-specific resources that would help understand the evolving IoT simulator field comprehensively. The following points are important to interpret the PRISMA flow diagram:

### 4.1. Research Question

The specific research questions addressed by this literature review are:

- Q1 (Section 5). In which macro-categories can we ascribe each IoT simulator regardless of its specific use case of interest?
- Q2 (Section 6). Do the specifics of each simulator usage scenario limit its generalizability, extension, or adaptability to other contexts?
- Q3 (Section 7). What sort of evaluation criteria are applied to measure the performance of the IoT simulator?
- Q4 (Section 8). Are there open challenges in IoT simulation still to be addressed?

### 4.2. Search Process

This literature review represents the results of a search of the papers published since 2014 and is available at the time of the writing (2023). This literature review was conducted by an initial search of the selected databases: Google Scholar, Science Direct, Sage journals, Wiley online library, ACM Digital Library, IEEE Xplore, Springer Link, Scopus, and Elsevier.

### 4.3. Search Keywords

We obtained the search keywords from our research questions to find the relevant literature. The keywords are applied in this review are: IoT simulators, IoT applications, IoT challenges, IoT simulators performance, and IoT simulators types.

### 4.4. Inclusion and Exclusion Criteria

The following criteria are defined to include the identified relevant articles in this review:

- The articles are published in the mentioned databases to maintain and ensure their quality.
- The articles focus on simulating and modeling IoT environments.
- Actively Maintained: The simulator must be actively maintained, with evidence of recent updates or releases.
- IoT Device and Network modeling: The simulator must be capable of modeling IoT devices and networks.
- Programming Language: We considered simulators available in various programming languages, including Java, C/C++, Python, and specialized IoT development languages.
- Open-Source: Only open-source simulators were included to provide a comprehensive view of the landscape.
- Ensure all articles are available online for easy access.
- Articles must be written in English.
- Only include papers that have undergone peer review.

Papers were excluded based on the following criteria:

- Any articles not aligning with the inclusion criteria.
- Articles focusing on IoT applications without addressing simulation and modeling systems, as they fall outside the scope of this literature review.
- Obsolete or Inactive: Simulators that were no longer maintained or had become obsolete were excluded.

Applying these inclusion and exclusion criteria ensured that our literature review focused on contemporary and relevant IoT simulators, providing a holistic view of the IoT simulation landscape and serving as a valuable resource for researchers, developers, and IoT enthusiasts.

### 4.5. Quality Assessment

The reliability of the search outcomes is verified through the analysis of information gathered from designated digital libraries. The authors review the abstracts listed in the search results to determine which papers should be included or excluded for further consideration. It has been observed that a majority of the studies assess synthetic datasets; however, our selection emphasizes papers based on real datasets within actual system environments to ensure practical relevance in engineering. A total of 58 papers met the selection criteria and were included in our literature review. The papers selected by the authors were collaboratively reviewed, and any discrepancies in opinions regarding the papers were resolved through discussion. Once the list was finalized, an in-depth analysis of the papers was conducted. The outcomes of our review and our contributions are detailed in the subsequent section.

## 5. Results of the Systematic Literature Review

This section overviews current IoT simulators and provides a detailed overall description. The following section focuses only on simulators that have been published since 2014 until this survey was written. Table 1 presents the existing IoT simulators including the focus, key features, and limitations of each simulator.

### 5.1. Current Simulation Platforms

IoTNetSim [24], is an advanced platform for modeling and simulating end-to-end Internet of Things (IoT) services and networks. It is a valuable tool for researchers and practitioners, offering a self-contained, multi-layered architecture to model IoT systems with different structures, application models, services, and network connections. Its distinguishing feature is the detailed modeling of IoT nodes and sensors, including power sources and mobility, enabling highly accurate simulations for testing configurations and algorithms. The platform covers a wide spectrum, from IoT networking, including wired and wireless connections and protocols, to services and applications, and is designed to be modular and extendable. It supports a broad spectrum of IoT components, including mobile nodes and gateways, facilitating the simulation of diverse IoT applications like environmental monitoring and disaster response. This platform enables realistic modeling across physical, networking, and application layers, incorporating cloud, fog, and edge computing models to simulate data flow and processing. IoTNetSim is equipped to model various protocols, including cellular, WiFi, LoRa, and ZigBee, enhancing studies on system performance. Its detailed simulation capabilities also hint at potential applications in creating virtual urban platforms, and exploring urban IoT ecosystems. Although not explicitly mentioned, the platform’s architecture suggests it could simulate SIoT-specific communication strategies. IoTNetSim emphasizes realistic simulations with features for simulating network and battery failures, highlighting its scalability for large-scale IoT infrastructures. Its modular design indicates compatibility with numerous IoT development tools and programming languages, making it a versatile tool for IoT system research and development. Real-world application examples, such as monitoring natural environments and responding to disasters, showcase IoTNetSim’s utility in guiding users from conceptual design to detailed evaluation of complex IoT systems. While it demonstrates its prowess in facilitating the evaluation of large-scale systems, it currently has limitations in supporting certain sensor types due to the complexity of modeling their mobility.

The large-scale NB-IoT (Narrowband Internet of Things) simulator described in [25] is a robust machine-to-machine traffic simulator tailored for studying IoT application performance in extensive environments like smart cities. Its unique aspect is integrating real geographical data from smart city open data projects, creating a virtual urban landscape where devices interact with the telecommunications infrastructure. This approach enhances the realism of IoT application simulations and aids in understanding their performance and potential issues. It adeptly simulates a wide array of IoT components, including NB-IoT and LTE devices, capturing their interactions, energy consumption, and mobility within urban ecosystems. This simulator enhances realistic modeling of IoT applications, from utility monitoring to urban mobility, by integrating real-world geographical data, allowing for precise device positioning within the simulated city. Its multi-layered architecture focuses on connection procedures and packet transmissions, efficiently simulating extensive networks without sacrificing speed or accuracy. Tailored for NB-IoT and LTE technologies, it aligns with 3GPP specifications, offering insights into network efficiency and scalability. The simulator’s systematic approach in creating a virtual urban platform starts from real geographical data collection, facilitating the simulation of device interactions across the urban environment. While focusing on NB-IoT and LTE, its architecture suggests flexibility in supporting various IoT protocols and standards, showcasing exceptional scalability for city-wide simulations with numerous eNodeBs and UEs through a discrete-event simulation approach. Programmed in Python, it is compatible with other IoT development tools, encouraging integration with data analysis algorithms. The simulator provides a robust framework for examining IoT application performance in urban environments, enabling scenario creation that reflects the complex network behavior at the city scale. However, since the simulator’s focus on NB-IoT and LTE (Long Term Evolution technology) devices and its physical layer abstraction limit, its applicability to other IoT device types and communication protocols potentially provides an incomplete perspective on IoT application behavior in real-world scenarios.

ASSIST (Agent Simulator for Social Smart Things), outlined in [26], creates a unique simulation tool for the Internet of Things (IoT), aiming to establish a Social Network-inspired environment for IoT entities. It empowers users to define smart Internet of Everything (IoE) entities, characterize their attributes, and establish social connections among them. ASSIST’s foundation is a cognitive middleware containing a Social Network of Agents, including SIoT Agents and a Broker Agent, representing the instantiated IoE entities. The SIoT Agents are autonomous, intelligent entities that employ ontologies for knowledge representation and employ a Publish/Subscribe pattern for service exposure and resource consumption. The Broker Agent oversees communication between SIoT Agents and manages their status. The simulator employs a flooding mechanism for SIoT Agents to find required services and a deterministic approach to forming social IoT clusters, distinguishing it from probabilistic methods. It integrates diverse IoT components like sensors and actuators as agents, allowing for rich interactions and social connections based on shared goals or ownership. The simulator excels in modeling IoT applications across scenarios, from environmental monitoring to energy management, using ontologies for knowledge representation and supporting dynamic social connections through a Publish/Subscribe pattern. ASSIST accommodates common IoT communication protocols such as CoAP, MQTT, and HTTP, suggesting its utility in virtual urban platform development and smart city solutions. It adheres to IoT standards and employs semantic web technologies for interoperability, testing IoT environments under various conditions to assess network resilience. Demonstrating scalability, ASSIST can manage extensive networks, indicating compatibility with semantic web technologies and various IoT development tools. A use case involving cultural heritage protection through WSNs, UAVs, and anomaly detection apps showcases its capability to simulate complex social interactions among IoT devices for effective service discovery and collaboration. However, it primarily supports Social Internet of Things (SIoT) environment simulation and SIoT Agent behavior.

GVSoC [27] is an event-driven simulator tailored for RISC-V-based IoT processors, offering a balance between accuracy and speed. It combines efficient C++ models with flexible Python configuration scripts, making it a valuable tool for researchers focusing on highly parallel and heterogeneous RISC-V-based IoT processors. Unlike the slow but accurate cycle-by-cycle simulators or the fast but less informative simulators, GVSoC delivers reasonably fast simulation with accuracy, enabling rapid exploration of diverse configurations, crucial for Design Space Exploration (DSE). It is highly configurable and flexible, with open-source availability for the research community. It supports a comprehensive range of IoT components, including processors and peripherals, enabling the simulation of complex systems with multicore and multi-memory levels. GVSoC excels in IoT application modeling, particularly for evaluating performance and energy efficiency in applications using deep neural networks and near-sensor data analytics. Its multi-layered architecture facilitates high-fidelity modeling of system components, including detailed simulations of the PULP platform. While focusing on structural and functional capabilities, GVSoC implies support for various communication protocols and standards, essential for architectural exploration. Its simulation features are particularly suited for Design Space Exploration, offering rapid configuration exploration with a significant speed-up over cycle-accurate simulations, and maintaining errors typically below 10 percent. GVSoC’s scalable architecture supports extensive IoT systems simulation, adaptable for urban infrastructure modeling. Utilizing C++ and Python, it provides a flexible and open-source development environment conducive to community research and IoT processor simulation. Documentation showcases real-life application examples, underscoring GVSoC’s role in accurate performance estimation and design exploration for low-power IoT system design. However, it currently focuses on RISC-V processors, lacking support for other architectures like ARM or MIPS, and its designed for near-sensor data analytics applications, including Deep Neural Networks (DNNs).

LoRa-MAB [28] is a versatile Python-based simulator tailored for decentralized learning resource allocation within IoT networks, specifically focusing on LoRaWAN applications. It employs an event-driven simulation framework using the Simply library to replicate LoRa link behavior across various network scenarios, including the capture effect and inter-SF collision. After a simulation, the simulator provides valuable insights into the network’s packet delivery ratio and overall energy consumption. LoRaWAN, a low-power, wide-area network technology commonly employed in IoT applications, constitutes the simulation’s primary area. This technology encompasses end devices, gateways, and network servers, forming a star-of-stars network topology. Gateways connect to the network server through IP-based backhaul networks, and LoRa modulation enables energy-efficient, long-range communication. Additionally, the LoRaWAN protocol offers security features such as encryption and authentication to safeguard data integrity and privacy.

MyiFogSim [29] is a virtual machine (VM) migration simulator in fog computing that extends the iFogSim simulator. The simulator is designed to support VM migration policies for mobile users, which involves migrating VMs to cloudlets based on user position. This approach can result in lower latencies and better quality of experience (QoE) for users. MyiFogSim incorporates a range of new classes, notably the Coordinate class that depicts map coordinates on a Cartesian plane, and the ApDevice class, an extension of the FogDevice class from iFogSim, equipped with the functionalities and duties of a wireless network access point. Additionally, the simulator introduces a VM migration strategy for mobile users within a fog computing environment, contrasting it with a scenario lacking VM migration. The outcomes of the simulations indicate that implementing the migration policy can lead to reduced latency compared to a setup that does not employ the migration policy.

The co-simulator discussed in [30] is a versatile platform developed for assessing the impact of emerging technologies on smart grids. It integrates two established simulators, Gridlab-D for power systems and CORE for communication networks. To tackle synchronization and interaction challenges between these components, the co-simulator employs a Graphic User Interface (GUI) for efficiency, software emulation for fidelity, and an Ethernet-tunnel-based distributed module for scalability. Co-simulator serves as a robust instrument for utility companies and policymakers to implement new IoT devices or strategies in upcoming smart grid frameworks. This platform offers flexibility and scalability, enabling experiments across various scenarios. It can run Linux applications on virtual nodes, using lightweight virtualization, and supports real-time and non-real-time modes, making it a valuable tool for studying the effects of smart grid technologies, particularly when developing a new simulator from scratch is complex and time-consuming.

The simulation platform presented in [31] offers an efficient Java-based solution for simulating large-scale IoT systems in urban settings. Its strength lies in simulating thousands of geographically distributed devices, a crucial aspect of extensive IoT deployments that require rigorous testing. Unlike traditional IoT simulators focused on low-level networking, this platform provides a high level of generality, modeling devices with multiple network interfaces and various mobility, network, and energy consumption patterns. This encourages code reuse and efficient development. However, it primarily concentrates on the application-layer perspective of IoT systems, which may limit its suitability for testing low-level networking aspects, as it abstracts device interactions more than specific data transmission and routing details.

The IoT software infrastructure proposed in [22] facilitates energy management and simulation in a city district by enabling the integration of diverse data sources and IoT devices. It incorporates real-time building energy profiles, environmental sensor data, and building/grid models, allowing for comprehensive energy consumption monitoring and management. The platform employs REST-based request/response and MQTT-based publish/subscribe communication paradigms, leveraging the LinkSmart OpenSource Middleware. It excels in modeling real-world IoT devices and ICT systems, integrating heterogeneous data sources, and simulating energy policies for district-level energy optimization while following the microservices paradigm. Nevertheless, further expansion is required to support additional IoT devices and data sources, like smart meters, weather sensors, and traffic sensors.

The dynamic co-simulation of Internet-of-Things (IoT) components using a multi-agent system [16] is an innovative approach for simulating complex IoT systems in a modular and adaptable manner. This approach enables the separate simulation of IoT components in different simulation tools, with agents representing each component, allowing them to join a running co-simulation dynamically. The connection between agents and simulation tools is facilitated through an interface concept. While promising, this approach involves using multiple simulation tools and the development of agents for each IoT component, which can be complex and time-consuming. Additionally, it lacks a complete solution for adding intelligence to the models, as it mainly optimizes the simulation process by reducing message traffic rather than enhancing agent intelligence.

RelIoT [32] is a framework for end-to-end reliability simulation in IoT networks, focusing on energy efficiency and reliability optimization. It integrates power, performance, temperature, and reliability modules into the widely used ns-3 network simulator. This framework estimates device temperature and reliability, a unique feature compared to other network simulators. It aims to balance energy consumption and Quality-of-Service (QoS) constraints, considering reliability as a design parameter that can be optimized. It offers runtime adjustments for the trade-off between performance and reliability and has minimal performance overhead for scalability. While validated with real-world experiments, RelIoT could benefit from support for more complex reliability models and diverse device types and applications, particularly those in challenging environments.

The MoSIoT framework [33] is an innovative approach designed for modeling and simulating Internet of Things (IoT) healthcare monitoring systems, particularly for people with disabilities. It utilizes the principles of model-driven engineering (MDE) to facilitate the creation of customizable and efficient healthcare monitoring scenarios. This framework allows healthcare professionals to easily simulate complete IoT healthcare scenarios, tailored for various disabilities and diseases, and subsequently generate final IoT systems. Key features of MoSIoT include a set of models for scenario simulation, integration with enterprise cloud architecture for data simulation, and support for commercial IoT hubs like Azure IoT Central. The framework’s emphasis on customization, ease of use for non-technical users, and its potential for improving remote healthcare monitoring makes it a significant contribution to the field of IoT and healthcare technology.

The hybrid simulation-based testing approach in [23] combines simulation and real-life testing to evaluate large-scale IoT applications, aiming to effectively simulate the interactions between local entities (LEs). It utilizes the IEEE-standardized Parallel and Distributed Simulation (PADS) methodology, particularly implemented through the Gaia/Artis IoT simulator, which can run thousands of virtual LEs in parallel. The main challenge addressed is the scalability required to manage real-time interactions among numerous LEs, achieved partly through cloud-based infrastructure and efficient synchronization mechanisms between real-life and simulation environments. However, the approach is primarily focused on testing large-scale Internet of Things (IoT) applications at the system level, and its main goal is to effectively facilitate interactions between local entities (LEs) in large IoT environments. So, it does not directly impact the use of renewable energy sources.

SimulateIoT [34] is a Domain Specific Language (DSL) that streamlines the design, code generation, and execution of IoT simulation environments. It offers time and cost savings for developers by enabling the creation of scalable IoT systems without substantial hardware and software investments. Developers define models with numerous Node elements, generating code that adheres to recognized software architecture patterns like publish-subscribe and Docker containers. SimulateIoT’s flexibility allows for easy adaptation to different target technologies. Key features include Model-Driven Development for handling IoT system complexity, the ability to define various nodes and policies with granularity values, and support for both CloudNode and FogNode. However, the node mobility in this simulator has been partially developed, and the hardware simulation is only managed by the size attribute at ProcessNode, which implies several constraints to avoid creating specific software elements. Second, the current version of the simulator IoT environment only allows defining connected nodes by TCP/IP, and it assumes that connectivity is guaranteed.

SimulateIoT-FIWARE [35] is a domain-specific language (DSL) that enables the design, code generation, and execution of IoT simulation environments on the FIWARE platform. The language is based on the SimulateIoT metamodel [34], which defines the concepts and relationships required to model IoT systems. SimulateIoT-FIWARE extends the SimulateIoT to include FIWARE-specific concepts and relationships, such as context brokers, IoT agents, and IoT devices. The language also includes a set of M2T transformations that generate code for specific FIWARE technology, such as Orion Context Broker and IoT Agents. SimulateIoT-FIWARE is flexible and scalable and based on open-source technology, allowing developers to choose the necessary components and integrate them with other technologies as required. However, SimulateIoT-FIWARE is tailored to the FIWARE platform, which may limit its applicability to other IoT platforms.

EdgeCloudSim [36] is a simulation framework tailored for evaluating IoT services over Edge and Cloud systems. It excels in its fine-grained analysis, covering service time, resource utilization, energy consumption, and service reliability. Notably, it accommodates mobile devices with diverse hardware characteristics, expanding its applicability. The framework stands out for its dual consideration of computational and network aspects, encompassing WLAN and WAN communication models, device mobility, load generation, and virtual machine usage models. EdgeCloudSim’s capacity to detail IoT service provisioning and the Edge/Cloud trade-off empowers researchers and practitioners. Furthermore, it introduces novel functionalities, like dynamic device hardware configuration, improved output, and expanded service simulation. However, its limitation lies in potentially missing nuances of diverse hardware features, potentially leading to inaccuracies in simulation results.

IoTSim-Edge [37] is a simulation framework addressing IoT and edge computing challenges. It enables data-driven decision-making in smart environments like homes and transport. The framework models various aspects, including device diversity, communication protocols, mobility, and battery features. It supports mobile IoT devices and handles handoffs between edges for consistent communication. IoTSim-Edge is built on existing simulators, capturing the complete behavior of IoT and edge computing systems. It allows for modeling various IoT protocols and their energy consumption profiles and provides a new abstraction for IoT application graph modeling. However, it does not consider the energy consumption of the entire IoT and edge computing infrastructure.

SimulateIoT-Mobile, detailed in [38], is a model-driven development tool to simplify the simulation of complex Internet of Things (IoT) environments with mobile nodes. Developers can employ it to model, validate, generate, and simulate IoT systems with mobility characteristics. The tool’s metamodel, built on the Eclipse modeling Framework (EMF), defines IoT system structure, while the Graphical Concrete Syntax provides a visual representation for enhanced understanding. This tool is an extension of SimulateIoT [34] and effectively addresses the absence of mobile node modeling capabilities. It utilizes the MQTT protocol for publish/subscribe communication and integrates an MQTT mobility management model to handle mobility within IoT systems. However, the tool has limitations, such as the assumption of guaranteed connectivity, which may not reflect real-world conditions where connectivity can be intermittent or disrupted.

PIoT [39] is a large-scale simulator designed to assess the network performance of IoT devices in a city-wide context. It includes a front-end for simulation configuration, a back-end for modeling millions of IoT devices using cellular infrastructure, and geographical and application databases. The architecture employs a grid structure based on a realistic city model and supports various elements like NB-IoT, network slicing, MEC, and beamforming. PIoT enables the testing of applications and KPI data extraction for AI and ML algorithm development. However, its primary focus is on network performance evaluation, with less emphasis on IoT device energy sources.

ABS-SmartComAgri [5] is a novel simulator for smart communication protocols in the context of precision agriculture. This is an open-source, agent-based tool designed to efficiently manage pesticide usage in agriculture by implementing smart communication protocols. It allows the simulation of various strategies, including broadcast, neighbor, and low-cost neighbor protocols, to optimize electric power, crop health, pesticide consumption, and overall network performance. The simulator is unique in its application to precision agriculture, explicitly targeting the reduction in pesticide usage and energy consumption while maintaining crop health. It is a valuable tool for testing and developing communication protocols before deploying them in real-world wireless sensor networks, contributing significantly to advancing smart agricultural practices.

FS-IIoTSim [40] is an innovative network simulation framework specifically designed for the performance evaluation of Industrial Internet of Things (IIoT) systems. Characterized by its flexibility and scalability, the tool adeptly supports both existing and modified communication protocols, addressing the complexities inherent in IIoT environments. FS-IIoTSim is structured into three integral components: scenario modeling, performance evaluation, and a user interface. Scenario modeling facilitates the creation of detailed industrial sensor network models and the generation of trace files containing device operation logs. The performance evaluation component analyzes these trace files, focusing on key metrics such as throughput, latency, and energy consumption.

IoTSim-Osmosis, as detailed in [20], is an advanced simulation framework designed for deploying Internet of Things (IoT) applications within integrated edge-cloud environments. Rooted in osmotic computing principles, it enables dynamic workload transfer between cloud data centers and edge devices, driven by performance and security triggers. This framework uniquely caters to the complexities of IoT applications and the heterogeneity present in integrated edge-cloud environments. Notably, it is the first framework to offer unified modeling and simulation for intricate IoT applications in such diverse settings. While existing frameworks support cloud-edge integration, none can directly accommodate osmotic computing due to IoT application intricacies and environmental diversity. IoTSim-Osmosis empowers researchers to evaluate end-to-end IoT application performance using osmotic computing concepts comprehensively. A case study on electricity management and billing demonstrate its potential. However, the framework has limitations, including expanding the wireless communication layer and incorporating support for security and privacy simulation. Assuming fixed IoT device locations impacts accuracy, necessitating further research to address varying signal factors and mobility. Additionally, research efforts are required to develop models and algorithms for security and privacy simulation.

As an extension of IoTSim-Osmosis, IoTSim-Osmosis-RES [2] encompasses a range of features pertinent to sustainable and autonomic IoT ecosystems, offering an assessment of various factors such as solar radiation levels, the utilization of renewable energy sources, the adoption of low-emission sources, and the battery capacity of IoT devices. It aims to simulate osmotic computations within the context of renewable energy sources and autonomic agents, thereby facilitating the analysis of distributed management algorithms. IoTSim-Osmosis-RES distinguishes itself as a unique IoT simulator capable of encompassing energy management, diverse power sources, and network infrastructure. In contrast to other simulators, it can simulate fluctuating weather conditions and the utilization of renewable energy sources, while also affording an easily extensible system that empowers researchers to specify their own virtual machine (VM) and power management policies. This framework is further characterized by its support for Software-Defined Networking (SDN), IoT devices, IoT device batteries, and renewable energy sources. This simulator has been linked with traffic simulation to simulate VANET environments in [4].

SimulatorBridger [4] is a novel simulator extended IoTSim-Osmosis-RES to bridge IoT simulation with traffic simulation. This simulator integrates IoTSim-OsmosisRES, an IoT simulator, with SUMO, a traffic simulator, to enable realistic simulation of Vehicular Adhoc Networks (VANETs) in urban mobility scenarios. The key innovation lies in the simulator’s ability to handle dynamic and heterogeneous environments by managing the mobility of vehicles and the communications of IoT devices. SimulatorBridger tackles the gap in current VANET research that lacks a simulation framework focused on IoT infrastructure. It facilitates seamless integration of mobility, IoT devices, diverse technologies, and battery management within dynamic settings. This simulator addresses the difficulty of replicating the behavior of highly mobile nodes within traffic networks that have constrained energy resources and the absence of centralized control. It allows for the evaluation of energy efficiency in vehicles and communication within VANETs, utilizing IoT infrastructure to enhance both realism and efficiency. However, the simulator does not support direct communications between IoT devices. Authors highlight their future works to extend the simulator’s applications, including load balancing in traffic lights and accommodating new technologies like 5G in VANETs.

### 5.2. Classifications of IoT Simulators

IoT simulators are categorized into distinct types based on their operational level within the overarching IoT architecture and the specific location where both data processing and simulation tasks are executed, into cloud, fog, and IoT device simulators [41]. Table 2 shows these three categories and some example simulators for each one.

#### 5.2.1. Cloud Simulators

Cloud IoT simulators primarily emulate cloud-based IoT platforms and services. Their design is rooted in understanding the intricate interactions between IoT devices and cloud services. They help evaluate the performance and efficiency of cloud resources, scheduling algorithms, and application deployments. This includes the mechanisms of data storage and processing in the cloud, as well as the intricacies of cloud-based analytics. Typically focuses on large-scale simulations involving multiple data centers and thousands to millions of VMs. They are used to evaluate cloud service models (IaaS, PaaS, SaaS), resource allocation strategies, and energy efficiency. Simulators of cloud computing emphasize the complexity of data centers, virtualization, and the management of large-scale resources. Examples of such simulators include the CloudSim Simulator. The foundation of these simulators lies in the services provided by cloud platforms, encompassing aspects like device management, data storage, analytics, and machine learning. The following are some examples of cloud simulators:

- IOTSim [37] is a versatile simulator implemented on top of CloudSim, primarily focused on modeling and simulating multiple IoT applications in shared cloud data centers. It covers various modules, including IoT application modeling, MapReduce data processing, cloud data center management, and network simulation. It offers a layered architecture that facilitates the simulation of various IoT components, including VMs on data center nodes with diverse hardware configurations, enabling detailed modeling of IoT applications from smart cities to healthcare systems with big data technologies like MapReduce. IOTSim excels in processing large volumes of data using parallel processing technologies, simulating network and storage delays critical for IoT applications, thereby providing a realistic model for the execution of parallel and distributed applications. Its protocols are tailored for efficient big data processing in cloud computing environments, supporting both batch and stream models, and demonstrating scalability and adaptability for complex IoT environments. The architecture integrates CloudSim’s core engine with layers for big data and user code, enhancing the simulation of network and storage delays. IOTSim’s design is suited for a range of IoT applications, from building automation to wearable tech, highlighting its ability to simulate batch-oriented applications with high accuracy and offering a cost-effective solution for IoT solution development in cloud computing environments. IOTSim excels in accurately simulating IoT application performance and resource scalability in cloud environments but lacks support for stream-oriented IoT applications.
- SimIoT [42] is a simulation toolkit designed to analyze cloud computing systems in the context of IoT. It extends SimIC by including user submissions from IoT devices like sensors and smartphones. It models various entities and interactions, including users, data centers, and virtual machines, with a focus on real-time constraints. The toolkit offers message-exchanging optimization and supports heterogeneity, making it versatile for various cloud configurations. Although valuable for healthcare and information processing scenarios, SimIoT lacks a real-world IoT implementation, physical device simulation, mobility support, and explicit energy efficiency measures.

#### 5.2.2. Edge Simulators

Edge simulators specifically focus on the computing resources that are located at the edge of the network, closer to the source of data generation, which are IoT devices. Unlike cloud or fog computing, edge computing aims to process data at or near the source, significantly reducing latency and bandwidth use. These simulators are crucial for evaluating the performance, reliability, and operational efficiency of edge computing environments. They model the dynamics of data processing, storage, and application execution at the network’s edge, enabling researchers and developers to explore various edge deployment scenarios, resource management strategies, and application behaviors in real-time conditions. Edge simulators are designed to address challenges such as limited computing resources, network connectivity variations, and the seamless integration of edge computing with cloud and fog layers. This allows for the optimization of IoT applications and services that require immediate data processing and decision-making capabilities. Examples of edge computing simulators include EdgeCloudSim and IoTSim-Edge, which offer detailed modeling tools for edge computing scenarios, from single-edge nodes to complex, multi-tier architectures involving edge and cloud collaborations. The following are some examples of edge simulators:

- CupCarbon, introduced in [43], is a cutting-edge platform for designing and simulating IoT Wireless Sensor Networks (SCI-WSN) in Smart Cities. It addresses the increasing prevalence of radio communication systems and technological advancements enabling IoT. CupCarbon stands out from traditional simulators by offering realistic modeling of radio channels and interferences, accounting for deployment environments, supporting mobile nodes, and enabling behavioral analysis in practical scenarios. It is a versatile tool with a multi-agent environment for mobility scenarios and event generation. It offers realistic modeling of radio channels and interferences, supports dynamic mobile nodes, and includes a comprehensive range of IoT components such as microcontrollers, sensors, batteries, and radio modules optimized for energy efficiency. The platform supports ZigBee 802.15.4 for WSN applications, and long-distance communication technologies like LoRa and SigFox, facilitating direct communication to base stations and reducing the need for extensive sensor node networks. CupCarbon’s novel architecture integrates environmental factors, mobility, and accurate radio propagation models for urban settings, enhancing network deployment studies and interference detection. It supports physical layer communication standards (ZigBee, WiFi, LoRa) for evaluating link quality and transmission conditions. The simulator’s 2D/3D virtual urban platform aids in planning smart city projects by simulating sensor network deployments, node mobility, and radio propagation. With its discrete event simulation kernel, CupCarbon scales to simulate dense sensor networks, crucial for future smart cities. Its modular, Java-based design ensures compatibility with various IoT development tools, facilitating customization and integration. CupCarbon’s utility in simulating smart city environments, showcasing sensor deployment, mobility modeling, and communication link visualization, underscores its comprehensive toolset for designing, simulating, and visualizing IoT network dynamics in urban settings. Although it represents a significant advancement in wireless sensor network simulation for IoT and Smart City applications, it has limitations, including integrating only two radio propagation models and potential challenges with complex scenarios.
- Contiki-Cooja [44] is a network simulation tool derived from the Contiki operating system. Developed in Java, it enables users to specify both large and small Contiki motes (sensor network nodes) for deployment across the network. After running a simulation, users can access crucial network data, including mote outputs and timelines. It is worth noting that motes can be custom-defined using template options. These simulations primarily emphasize hardware and network challenges rather than IoT scenarios or communication models like publish-subscribe.
- EdgeMiningSim in [45], offers a simulation-driven approach for IoT data mining within edge computing. This methodology addresses IoT applications’ unique challenges in data mining, aiming to empower domain experts with actionable insights for decision-making in dynamic IoT scenarios. The simulator is adaptable to various IoT applications, encompassing algorithmic, infrastructural, and contextual aspects often studied in isolation. It employs an interactive and iterative approach, balancing technical objectives and business interests. It simulates a diverse IoT ecosystem, including sensors and edge servers, integrating algorithmic, infrastructural, and contextual aspects for a comprehensive architectural design, network modeling, and topology definition. Employing a multi-layered architecture, EdgeMiningSim offers realistic modeling of device and application characteristics, such as mobility and energy consumption. While not extensively detailed, the simulator accommodates various IoT communication protocols and strategies, including task offloading and edge server management. It highlights the role of simulation in the early stages of IoT deployment, supporting the planning and evaluation of virtual urban platforms. The simulator includes mechanisms like flooding for efficient information dissemination among nodes and supports lightweight, decentralized data processing techniques. Demonstrating high scalability, EdgeMiningSim is suitable for large-scale IoT deployments, with a modular architecture indicating compatibility with numerous development tools. Through a smart environmental monitoring case study, EdgeMiningSim exemplifies its ability to simulate real-life IoT data mining scenarios, bridging the gap between theory and practice, and underscoring its value for researchers and practitioners. While promising, EdgeMiningSim does require substantial computational resources, which may pose challenges for resource-constrained IoT devices with limited processing power and memory.
- Mercury [46] is an open-source framework tailored for simulating real-time fog computing scenarios, emphasizing low latency, high data throughput, and 5G capabilities. It provides a detailed structural and behavioral model, offering insights into edge infrastructure and quality of service (QoS) optimization. Mercury is particularly suitable for data stream analytics applications and federated computation offloading. However, it is important to note that in the first approach of the scenario under study, cloud computing is not included in Mercury’s fog model. The framework is primarily focused on investigating how location awareness and mobility impact the Quality of Service (QoS) and operational costs. This analysis aids in optimizing both the sizing and functioning of edge infrastructure necessary for supporting services that rely heavily on computation offloading.

#### 5.2.3. IoT Simulators

IoT simulators simulate the behavior of IoT devices, networks, and services. They are used to evaluate the performance, scalability, and reliability of IoT systems. Their main features are device modeling, network communication, data generation and processing, etc. They are used to test IoT protocols, device-to-device communication, and scalability of IoT networks. Simulators of IoT computing focus on the heterogeneity of devices, network protocols, and scalability challenges. They are designed to handle many IoT devices, potentially in the order of millions, although not all IoT simulators simulate the cloud. Thus, comprehensive simulation environments must be developed that represent the entire journey of data from its origins in IoT devices to its eventual processing and storage in a data center in the cloud. The following are some examples of IoT simulators:

- In [47], the authors propose a multi-level IoT simulator designed for smart territories, emphasizing scalability and advanced modeling techniques. The approach aims to promote the development of sustainable services in non-metropolitan areas. The simulator offers a two-level simulation approach, encompassing IoT systems’ physical (sensors and actuators) and logical (software components) aspects. While the methodology shows promise in enhancing services for decentralized regions, it faces challenges in handling large-scale IoT environments. Its distinguishing feature is the comprehensive modeling of both physical and logical scenarios, essential for the development of sustainable services in non-metropolitan areas. Physical scenarios encompass the tangible components of IoT systems, such as sensors and actuators, which interact directly with the environment. An illustrative example is the implementation of a smart market, where various producers utilize these devices to advertise product availability, enabling customers to engage interactively with the marketplace in real time. This scenario demands precise simulation of physical interactions and device deployments, ensuring the efficient operation of sensors and actuators within a dynamic market environment. Conversely, logical scenarios address the software-driven aspects of IoT systems, including data processing algorithms, communication protocols, and decision-making mechanisms. These scenarios are pivotal for simulating the complex, intangible interactions that govern the behavior of IoT systems. In the context of the smart market, logical simulation involves modeling the publish/subscribe mechanisms that facilitate information exchange between producers and consumers. It also encompasses the detailed simulation of wireless communication protocols, crucial for providing customers with timely information on product availability and guiding them through the market. The multi-level simulation approach, integrating coarse-grained and fine-grained simulations, is instrumental in addressing the intricacies of such scenarios. At the coarse level (Level 0), the simulation provides a broad overview of the smart territory, encompassing general interactions and behaviors. The fine-grained level (Level 1) delves into detailed simulations of specific areas, such as the smart market, focusing on intricate wireless communications and proximity-based interactions. This dual-level methodology ensures scalability and maintains high levels of detail where necessary, thereby facilitating the intricate simulation of IoT environments for the advancement of smart services in decentralized regions. This simulator designed to support the development and optimization of smart services in cities and decentralized areas, incorporates a comprehensive range of IoT components such as sensors, RFID devices, and mobile terminals. It employs a two-level simulation approach: a coarse simulation for general behaviors and interactions, and a more detailed simulation for specific aspects like wireless communications, utilizing tools like OMNeT++. This simulator supports various protocols crucial for realistic IoT scenarios, such as epidemic dissemination protocols, and integrates tools like MASON and SUMO for modeling urban systems and intelligent traffic control. While not explicitly mentioning a flooding mechanism for Service IoT (SIoT) Agents, its flexible architecture suggests compatibility with various communication mechanisms. It addresses scalability challenges through adaptable programming frameworks, ensuring compatibility with a broad range of development tools. It is capable of modeling complex scenarios, like a smart market, illustrating its potential to significantly contribute to smart city services and strategies.
- Ref. [48] introduces a 3D virtual environment simulator for IoT-based smart house systems, enabling controlled testing and evaluation. The simulator features virtual sensors replicating real-world conditions and an autonomous agent generator to simulate human-like behavior in the smart house. It is a cost-effective tool for testing and evaluating IoT-based smart house systems, enhancing design and issue identification. It features a wide array of virtual sensors and an autonomous agent generator to accurately replicate real-world conditions and human-like behavior in smart houses. This simulator supports a broad range of IoT components, such as motion detection and temperature sensors, providing a comprehensive tool for architects and engineers to configure smart house layouts intuitively via a GUI-based interface. Its innovative motivation-based behavior planning method for generating autonomous agents ensures realistic interactions within smart environments, suggesting its potential use in larger urban planning projects. Although it does not specify communication protocols, its design implies compatibility with common IoT technologies. The GUI facilitates easy design and testing of complex systems, indicating scalability and offering insights into optimal sensor placement and smart house dynamics. The development environment is flexible, hinting at compatibility with various IoT tools and mainstream programming environments, enabling thorough testing and refinement of smart house systems before real-world implementation. However, further improvement in virtual sensor accuracy through machine learning is suggested.

#### 5.2.4. Fog Simulators

Fog computing is an extension of cloud computing that brings computation closer to the data source which are IoT devices. Fog simulators help in modeling and evaluating the performance of fog nodes, their interaction with cloud and edge devices, and the overall system efficiency. The main features here are hybrid modeling (combining features of cloud and IoT), latency evaluation, local data processing, etc. They are designed to handle a mix of large-scale cloud resources and numerous edge devices. They are used to evaluate data offloading strategies, fog node placement, and hybrid cloud-fog architectures. Simulators of fog computing deal with the complexity of integrating both cloud and edge components, ensuring seamless data flow and processing. Some of the important and widely used fog simulators are: FogTorch [49], EmuFog [50], EdgeCloudSim [36], FogNetSim++ [51], iFogSim [9], and YAFS [52]. Listed below are a few examples of fog simulators:

- MobIoTSim [53] is a mobile IoT device simulator designed to facilitate the learning, testing, and development of IoT applications more efficiently. It can emulate IoT devices, generate real-time sensor data, and respond to messages using popular IoT protocols and data formats. Users can create IoT environment simulations with custom settings and connect the simulator to cloud gateways, like IBM Bluemix, for device management and notifications. However, MobIoTSim may not fully replicate real IoT device behavior and lacks support for simulating network errors, recording and replaying cases, and connecting real devices. It also does not explicitly simulate device mobility. These additional features could enhance its realism and utility for IoT testing.
- iFogSim [9] is an academic toolkit focused on simulating resource management policies in IoT, Edge, and Fog computing environments. It evaluates policy impact on latency, energy use, and network congestion metrics. This toolkit can model complex fog environments, aiding the assessment of real-time IoT applications. iFogSim comprises four key components: application, network, resource models, and a simulation engine. It includes sample policies for reference but does not support mobility. This resource is valuable for academic research into IoT, Edge, and Fog computing, particularly regarding resource management policy assessment. However, iFogSim does not support mobility.

Therefore, while there are overlaps, each type of simulator is tailored to address the unique challenges and characteristics of its respective domain. Some of the differences between these simulators are related to the communication layers or assumptions concerning IoT/Edge/Cloud/Fog. For example, fog simulators primarily focus on layers 4 to 7 of the OSI model Figure 1, as these are most relevant to the data processing, application protocols, and communication management functions of fog computing. On the other hand, edge simulators such as SimulatorBridger [4], are only simulating layers 2 to 5 and layer 7 as Software Defined Networks Figure 1.

## 6. Applications of IoT Simulators

### IoT Applications

IoT applications cover a wide range of domains, each addressing specific challenges and requirements. In this section, we will discuss some of the current applications of the Internet of Things based on IoT simulators, including smart homes, healthcare, smart agriculture, transportation [4,54], smart cities, and smart industries. Smart services provided by the Internet of Things greatly benefit human life. Most activities can be performed anywhere, anytime, and provide instant decision-making for efficient management. As stated in [3], the most researched application is healthcare, followed by transportation and the environment. The other areas include utilities [55], military [56], safety [54], education [57], and financial [58].

- Smart Cities: IoT simulators are essential for urban planners and developers in crafting interconnected, intelligent urban environments. These simulators are instrumental in a range of applications, from traffic management to environmental monitoring, by leveraging data from diverse IoT devices such as vehicles [4], bicycles [8], and satellites [6]. In traffic management, IoT simulators use data from vehicles equipped with sensors to model and improve urban traffic flows, reducing congestion and enhancing road safety [4]. Bicycle-sharing systems in cities benefit from IoT simulators that analyze data from GPS-equipped bicycles [8]. These simulators help in understanding cycling patterns, identifying high-demand areas, and planning the expansion of cycling infrastructure. Satellite imagery and data play a crucial role in large-scale environmental monitoring, offering a macro perspective on urban development, green spaces, and pollution levels. IoT simulators that incorporate satellite data [6] can predict weather patterns, monitor air quality, and assess the impact of urban planning decisions on the environment. Hence, all the IoT simulator tools in smart city applications excel in modeling traffic management systems to alleviate congestion and enhance public transportation efficiency, while also optimizing waste collection by predicting bin fill levels, reducing operational costs. They enable adaptive street lighting, adjusting brightness based on pedestrian and vehicular movement for energy savings. Additionally, they are crucial in environmental monitoring, tracking pollution levels, and providing real-time weather updates for disaster preparedness. In the broader scope of smart city development, these simulators are adept at environment modeling, creating accurate digital representations of urban spaces, offering data analytics for informed decision-making, and facilitating emergency response planning [2,59].
- Smart Homes: In the domain of residential technology, IoT simulators play a pivotal role in envisioning the future of smart homes. They are instrumental in modeling energy consumption, enabling homeowners to comprehend and minimize their energy usage, thereby reducing their environmental impact and utility costs. A significant aspect of their application lies in testing home automation features, such as adaptive lighting and appliance control, for both user-friendliness and operational efficiency. This ensures seamless integration of these technologies into daily life. In the realm of smart home development, the focus areas include device integration, ensuring seamless connectivity and interoperability among various smart devices; user interaction, emphasizing intuitive interfaces and control mechanisms for an enhanced user experience; and robust security measures, which are essential for protecting sensitive data and maintaining resident privacy. These elements collectively contribute to the advancement of smart home technologies, optimizing both functionality and user engagement [20,48,60]. Additionally, these simulators are valuable in assessing home security systems and identifying potential vulnerabilities to safeguard against intrusions. For example, consider a smart building equipped with IoT sensors for monitoring temperature, occupancy, and security. These sensors are typically battery-powered to allow placement flexibility and reduce installation costs associated with wiring. An adversary targets these IoT devices with a battery drain attack, intending to disrupt the building’s monitoring capabilities.
- Healthcare: The integration of IoT in healthcare represents a significant transformation, particularly evident in the use of simulators for various applications. These simulators are crucial in modeling remote patient monitoring systems, which are essential for accurately tracking vital signs and health metrics, with built-in mechanisms to trigger alerts in case of anomalies. They also play a vital role in optimizing elderly care systems, and monitoring movement, activities, and health metrics to facilitate timely interventions. Additionally, medication adherence systems are tested for their efficacy in providing timely reminders and ensuring the consistency and accuracy of medication routines. In the broader context of healthcare simulation, key features include user interaction, which emphasizes intuitive interfaces for both healthcare professionals and patients to ensure efficient workflow and patient engagement. Security measures are of utmost importance to safeguard sensitive patient data and adhere to healthcare regulations. Data analytics is leveraged to extract meaningful insights from extensive medical data, enhancing patient care and informing research. Emergency response simulations are integral for training and preparing healthcare personnel to handle critical situations effectively. Furthermore, the simulation of medical procedures and Electronic Health Records (EHR) is vital for the education and training of medical professionals and for the testing and refinement of healthcare systems, ensuring their efficacy and reliability [7,33,61,62]. Consider a healthcare system where IoT devices are integral to monitoring medical equipment health, tracking patient care metrics, and regulating energy use. These devices constantly feed data into a central system, facilitating predictive maintenance of medical machinery and guaranteeing the highest standards of patient care. It is possible for attackers to result in rapid battery depletion or to falsify the sensor’s battery status. Consequently, medical equipment could run without proper oversight, potentially leading to costly malfunctions or, more critically, compromising the safety of patients.
- Agriculture: Modern agriculture heavily relies on technology, and IoT simulators play a crucial role here. Precision farming applications can be modeled to ensure that variables like soil moisture and crop health are accurately monitored, leading to optimized irrigation and fertilization schedules. When stimulated, livestock monitoring can help design systems that track the health, location, and movement of animals, ensuring their well-being and reducing losses. Agriculture simulators prioritize device integration, environment modeling, weather simulation, data analytics, precision farming, pest and disease simulation, and resource management. Device integration allows farmers to monitor and control various agricultural equipment and sensors. Environment modeling involves creating digital representations of farmland to analyze and optimize conditions. Weather simulation aids in assessing the impact of weather patterns on crop growth. Data analytics helps in making data-driven decisions for crop management and yield optimization. Precision farming involves the precise application of resources like water, fertilizers, and pesticides, increasing efficiency. Pest and disease simulation assists in identifying and mitigating threats to crops. Resource management ensures efficiently utilizing land, water, and other resources [3,5,63,64,65].
- Industrial IoT (IIoT): The industrial sector’s efficiency and productivity gains with IoT are significant. Predictive maintenance applications, when simulated, can help industries predict machinery wear and tear, reducing downtime and maintenance costs. Supply chain monitoring can be optimized to track goods and materials in real time, ensuring timely deliveries and reducing losses. Energy consumption optimization, when modeled, can lead to significant cost savings and reduced carbon footprints for industries. In the Industrial IoT domain, features center around device integration, sensor and network simulation, data analytics, predictive maintenance, security, and scalability. Device integration facilitates the connection and management of industrial devices and equipment. Sensor and network simulation allow the testing and optimization of IoT sensor networks. Data analytics leverages industrial data for predictive maintenance, optimizing machinery performance and minimizing downtime. Security measures are critical in safeguarding industrial processes and sensitive data. Consider a scenario in a smart industrial complex where IoT devices monitor machines, track inventory, and even manage access controls. These devices, often battery-powered to ensure uninterrupted service, are the lifeblood of operations, ensuring efficiency and safety. Within this complex, battery-operated IoT sensors are strategically placed on cargo containers to monitor temperature, ensuring that sensitive goods are stored optimally. These sensors relay real-time data to a central system, which makes necessary adjustments. Recognizing the critical nature of these sensors, cyber attackers target the battery management systems of these IoT devices. By exploiting vulnerabilities, they can either cause premature battery drainage or manipulate the sensors to report inaccurate battery levels. This could lead to goods being stored at incorrect temperatures due to perceived sensor outages or malfunctions, potentially resulting in significant financial losses [66]. In addition, scalability is crucial to ensure that IoT solutions can grow to accommodate expanding industrial operations [40,67].

When we compare IoT simulators from various domains Table 3, we observe that they emphasize various features differently. In smart home simulators, for example, IoTSim-Osmosis, the primary focus lies on user interaction, security, and energy efficiency. Smart city simulators such as IoTSim-OsmosisRES and SimulatorBridger are designed to excel in environment modeling and traffic and weather simulation. On the other hand, patient monitoring, medical procedures, and EHR simulation are vital features of IoT simulators in the healthcare domain. However, simulators in smart homes and smart cities do not support these features. Smart home and smart city simulations may have similarities focusing on energy efficiency, but they cannot be used in healthcare, since they do not support patient monitoring or medical procedures. In contrast, however, agriculture simulators prioritize weather simulation, precision farming, and pest and disease simulation, which is not able to support medical procedure features in a healthcare domain. Lastly, the central features in the industrial IoT domain include predictive maintenance, fault detection, and remote monitoring and control. This means that IoT simulators do not support other features from other domains like pest and disease simulation.

## 7. Assessment Metrics for IoT Simulator Performance and Comparative Analysis

### 7.1. Evaluation Criteria for IoT Simulator Performance

Evaluating the performance of an IoT simulator involves assessing its ability to model, replicate, and predict the behavior of real-world IoT devices and networks accurately and efficiently. The most critical evaluation criteria applied to measure the performance of an IoT simulator: scalability, realism, latency measurement, network topology flexibility, device behavior modeling, protocol support, resource consumption, interactivity, extensibility, integration with other tools, performance metrics reporting, ease of use, reliability and stability, mobility modeling, and environment modeling. In the next paragraph, we will discuss why these metrics are critical to evaluating IoT simulators.

The most important criterion is scalability. As IoT networks can consist of many devices, simulators must handle large-scale simulations without performance degradation. So, they need to be able to simulate thousands to millions of devices, maintain performance under increased load, and provide consistent results in large-scale scenarios. In the realism metric, a simulator primarily aims to mimic real-world scenarios. So, the closer the simulation is to reality, the more valuable its predictions. Hence, they need to accurately replicate device behaviors, real-world communication patterns, and potential device or network failures. Moreover, latency measurement is a critical aspect because, in IoT, timely data delivery can be crucial. So, simulators must measure and report latencies accurately. Therefore, IoT simulators must measure time delays in data transmission, account for network congestion, and provide insights into potential bottlenecks. Aside from that, network topology flexibility is an important factor since real-world IoT deployments have diverse network topologies. Simulators should support and easily switch between different configurations. Hence, supporting various network shapes (star, mesh, tree, etc.), easing of reconfiguring topologies, and modeling of multi-tiered architectures are necessary features in IoT simulators. Device behavior modeling is another criterion, IoT devices can have varied behaviors based on their roles, energy sources, and capabilities. So, simulators should be able to simulate different states (active, sleep, error), model energy consumption patterns, and replicate device-specific functions. Also, protocol support is an essential metric in evaluating IoT simulators. Different IoT solutions employ different communication protocols. Thus, a good simulator should be able to support a variety of IoT protocols. The inclusion of popular IoT protocols like MQTT, CoAP, and HTTP, the extensibility to add new protocols, and the accurate modeling of protocol behaviors are important capabilities for IoT simulators. Regarding the resource consumption parameter, a simulator’s efficiency can be gauged by the computational and memory resources it consumes. So, low CPU and RAM usage, optimization for different hardware configurations, and minimization of unnecessary overheads are important features in IoT simulators.

For the interactivity metric, the real-time interactions enable users to modify scenarios on-the-fly and observe outcomes. So, IoT simulators should be able to have live modification capabilities, real-time visualization of network status, and instant feedback mechanisms. Also, the extensibility aspect is important. Simulators can be future-proofed by adding new features without overhauling their core. As a result, IoT simulators should have a modular architecture, be able to support plugins and extensions, and be easy to integrate new features into. So, integration with other tools is a very important criterion, which is enhancing a simulator’s capabilities by interfacing with external software tools. Hence, IoT simulators should support integration with data analysis tools and visualization platforms and be compatible with standard data formats. Moreover, performance metrics reporting is another essential aspect of evaluating the IoT simulator. Users should obtain detailed insights into various performance aspects of the simulated network. Hence, simulators in IoT environments should support comprehensive reports on metrics like throughput, packet loss, energy consumption, metrics visualization, and exportable data formats. In addition, ease of use is an important factor since a user-friendly simulator ensures that users can focus on simulation tasks instead of attempting to master the software. So, it should have an intuitive graphical user interface (GUI), well-documented user manuals, and availability of tutorials or guides. Additionally, reliability and stability are important criteria of the simulator, as the simulator’s consistent performance ensures that the users can trust the results. As a result, simulator tools must have minimal software crashes, robust error-handling mechanisms, and stable performance under various scenarios. In addition to previous metrics, mobility modeling is an essential factor in IoT simulations. Simulating movement and its impact on connectivity is vital for moving IoT devices. Simulators should support accurate modeling of device trajectories, the impact of mobility on connection stability, and the simulation of varied speeds and patterns. Lastly, environment modeling is another important criterion. Environmental factors can influence IoT device performance, so modeling physical obstacles, interference sources, and varying signal strengths based on environmental conditions are important features in IoT simulators. In conclusion, each criterion’s depth varies based on the simulator’s purpose, targeted users, and the specific domain of IoT it addresses.

In the previous part, we answered one of our research questions, which is the evaluation criteria applied to measure the performance of the IoT simulators. We identified a broad range of evaluation criteria. However, for our comparison analysis part, we have chosen to focus on some of these criteria that we believe are highly relevant to the current and emerging trends in IoT simulation. The next section compares the most recent IoT simulators based on some of the evaluation criteria. We focus on the criteria that are challenged in this domain.

### 7.2. Comparison of IoT Simulators

The most recent and commonly used IoT simulators and information are included in Table 4. The table summarizes the studied IoT simulators, focusing on their programming languages, availability, scalability, and support for SDN, IoT device mobility, energy models, and renewable energy sources.

The column language means the programming language used to code the experiments. The availability metric categorized simulators as available (open-source) or commercial. The mobility indicates whether the simulator has support for moving IoT devices. The SDN column (SDN) indicates simulator support for Software-Defined Networking. The energy model feature identifies if there is already a model (for cost and/or energy) implemented or if the user can add it via built-in features. The scalability metric measures the simulator’s ability to scale and simulate a wide range of IoT devices and network sizes. The RES aspect focuses on the ability of the simulator to support renewable energy sources. A ✓ indicates that the simulator reports that metric, while ✗ indicates that the simulator does not.

From the table, we can see that all the studied IoT simulators are designed using Java. And out of nowhere, all our studied IoT simulators are open-source. Mobility support is a critical requirement for IoT applications and services, considering they are usually attached to users or devices that are moving between different access points at the edge of the communication infrastructure. Out of the simulators analyzed, only MyiFogSim, EdgeCloudSim, and SimulateIoT-Mobile, SimulatorBridger support mobility. On the other hand, IOTSim, SimulateIoT, CupCarbon, and IoTNetSim can partially model an IoT environment where nodes are moving throughout the system. For example, IoTNetSim has limitations in supporting certain sensor types due to the complexity of modeling their mobility.

Software-Defined Networking (SDN) presents an effective approach for managing dynamic network settings, particularly in scenarios involving numerous connected devices and varied applications. SDN employs software-based controllers or application programming interfaces (APIs) to interact with the underlying hardware infrastructure, guiding the flow of network traffic [20]. Among the simulators that were analyzed, IoTSim-Osmosis, IoTSim-OsmosisRES, and SimulatorBridger are the only ones that support SDN. As part of these simulators support SDN, it provides mechanisms for SD-WAN (Software-Defined Wide Area Networking) networking. In these simulators, IoT, edge, and cloud ecosystems can be integrated, and the edge can be virtualized as well as incorporating SDN-aware infrastructure. Similarly, a cloud data center can include hosts that are virtualized as well as networks that are aware of SDN. These three simulators holistically integrate all these components in order to provide researchers with the necessary support to evaluate the performance of an end-to-end IoT application using the osmotic computing concept. Using SDN, network switches can be centrally managed with fine-grained traffic management capabilities. Moreover, network elements can be dynamically programmed and controlled through a central controller [2].

For energy modeling support, iFogSim, IOTSim, CupCarbon, IoTNetSim, SimulateIoT, IoTSim-Osmosis, IoTSim-OsmosisRES, and SimulatorBridger are supporting energy models. While these simulators offer some form of energy modeling, their depth and focus vary. CupCarbon, focusing on wireless sensor networks in smart cities, offers detailed energy modeling for sensor nodes. iFogSim and IOTSim, on the other hand, provide energy models more suited for fog and cloud computing environments, respectively. EdgeCloudSim authors’ mentioned the support of energy consumption models for mobile and edge devices, as well as cloud data centers, as a feature that needs to be developed. In contrast, the IoTSim-Osmosis framework evaluates the energy consumption of IoT applications holistically, taking into account the energy usage of different components in the edge-cloud continuum. It is important to note that in one of their scenarios, they neglected the battery consumption of the IoT devices due to static data generation. In contrast, IoTSim-OsmosisRES supports only battery management for IoT devices without including battery support for edge devices. Despite this, IoTSim-OsmosisRES and SimulatorBridger support energy modeling for RES (renewable energy sources), such as photovoltaic panels, and enable the evaluation of AC (Autonomic Computing) algorithms for osmotic computing. AC can help manage the available renewable energy sources efficiently. They are able to assess various parameters, such as the level of solar radiation, usage of the RES, usage of low-emission sources, and the IoT device battery capacity. The framework also enables the evaluation of the impact of different adaptation algorithms on the performance of the IoT system in terms of utilizing renewable energy sources. As a result, IoTSim-OsmosisRES and SimulatorBridger are the only IoT simulators that supports RES.

From the IoT simulators under study, iFogSim, SimIoT, SimulateIoT, and EdgeCloudSim have limitations in simulating large-scale environments. The other IoT simulators studied support scalability at a variety of levels. SimulateIoT-Mobile is designed to model and simulate complex IoT environments, but no information or testing exists. However, IoTSim-Osmosis is designed to handle the heterogeneity of integrated edge-cloud environments and the complexity of IoT applications. The scalability of IoTSim-Osmosis is demonstrated in terms of time and memory consumption, but no large-scale scenario has been tested using this simulator. For IoTSim-OsmosisRES and SimulatorBridger, using historical data to realize the simulation environment can help improve the framework’s scalability by reducing the simulation’s computational overhead. Also, using decentralized management can help improve the framework’s scalability by distributing the management of the IoT system across multiple nodes. On the other hand, the NB-IoT Simulator provides the clearest statement regarding scalability among the studied IoT simulators. It is specifically designed to handle large-scale networks spanning over an entire city. Also, it can model hundreds of base stations and thousands of devices and is based on realistic databases obtained from smart city open data projects.

These existing IoT simulators present a variety of methodologies for evaluating the performance of IoT-based applications in various environments, including edge computing and fog computing. Various metrics and parameters are used in these simulators to measure aspects such as energy consumption, scalability, and overall system performance.

IOTSim and IoTSim-Osmosis focus on metrics like VM computing cost, network cost, battery consumption, and the dynamics of IoT devices within osmotic and conventional computing environments. SimulateIoT-Mobile emphasizes the impact of jitter on Quality of Service (QoS), indicating the importance of network quality in IoT applications. IoTNetSim and others like NB-IoT Simulator and SimulatorBridger provide insights into the simulation of network behaviors, battery usage, and the efficiency of data transmission among IoT devices.

Scalability testing across these simulators varies widely, from manipulating datacentre configurations and VM numbers in IOTSim to evaluating the performance of IoT applications based on the number of IoT devices and their operational dynamics in IoTSim-Osmosis. Energy consumption parameters also differ, with simulators like iFogSim comparing deployment on fog devices versus cloud data centers, while RES focuses on the utilization of renewable energy sources and energy-efficient data processing algorithms.

IoTSim-Edge, SimIoT, and EdgeCloudSim each bring unique perspectives on the simulation of edge computing environments, highlighting the challenges of managing limited resources such as computational power, battery life, and network bandwidth. CupCarbon, on the other hand, delves into the simulation of network behaviors and energy consumption, emphasizing the effects of sensor connectivity on battery life.

Given the heterogeneity in the datasets used by these simulators and the diversity of metrics measured, it becomes evident that making a quantitative comparison between them is not straightforward. Simulators have been designed with a specific focus on IoT applications across a variety of computing paradigms. As a result of the differences between the simulated environments, as well as the varying approaches taken to measure performance and efficiency, it is difficult to establish a uniform basis for comparison. Since each simulator provides valuable insights into certain aspects of IoT system performance, a direct quantitative comparison between them is not possible due to the differences in their datasets and metrics. It is clear from this diversity that there is a wide variety of considerations in IoT research and development, which highlights the need for generic simulation tools Section 8.2.1 to provide more versatile and integrated simulation solutions in the future.

While several IoT simulators are available, to the best of our knowledge, we have not found an IoT simulator that deals with security issues around the Internet of Things, specifically batter-draining attacks. This limitation in existing simulation capabilities strongly motivates the need for the development of a secure simulation framework Section 8.2.2, which would focus on the security aspects of IoT, especially concerning energy-related attacks. By incorporating the simulation of battery-draining attacks and other security threats, researchers and developers can gain valuable insights into the resilience of IoT systems against malicious actions, enabling the design of more robust and secure IoT solutions.

## 8. Challenges and Future Trends

### 8.1. Current Challenges

According to our analysis review, most IoT simulators are designed to support a particular scenario with their abstractions. Hence, no generic IoT simulator can simulate any scenario from any domain in the IoT world. Moreover, many challenges still need to be addressed in the field of Internet of Things simulation.

Mobility, an essential feature of IoT, is not comprehensively represented in many IoT simulators. Most tools offer limited mobility modeling capabilities, underscoring the urgent need for advanced simulators. These advanced simulators should be capable of accurately depicting the dynamic nature of IoT topologies, especially for complex simulation environments such as smart vehicles, wearables, and individuals on the move. Given the rising prevalence of mobile IoT devices, future simulators are anticipated to incorporate sophisticated mobility models. These models might leverage real-time data and predictive analytics to emulate intricate mobility patterns and scenarios. The development of advanced simulators that can more accurately and comprehensively represent IoT deployments’ dynamic and mobile nature is urgently required, perhaps integrating real-world mobility data or leveraging machine learning to predict mobility patterns.

Additionally, the recent integration of SDN into existing simulators highlights a gap in current research. As SDN and Network Function Virtualization (NFV) become integral to IoT networks, simulators must natively support these technologies, offering a holistic view of network dynamics and management. The absence of supporting these technologies in IoT simulators may prevent valuable insights into IoT paradigms centered on SDN. Given the increasing importance of SDN in modern network environments, researchers should prioritize its comprehensive representation in simulators, possibly by developing modules or plugins that can be integrated into existing tools.

Energy modeling is crucial to global sustainability efforts, yet many IoT simulators currently lack comprehensive energy models. This absence is particularly concerning given the pivotal role of energy consumption and management in real-world IoT deployments. Without these models, simulators may produce results that fail to accurately represent the energy dynamics of actual IoT devices and networks. Consequently, there’s a critical need for future simulators to not only focus on energy consumption but also on generation, storage, and optimization, with a particular emphasis on renewable energy sources. To the best of our knowledge, IoTSim-Osmosis-RES and its extended version: SimulatorBridger are the only IoT simulators that can simulate floating weather conditions, and renewable energy sources and provide easily extendable system that enables researchers to specify their own virtual machines and power management policies.

The scalability issue remains unresolved for a significant subset of simulators. In the IoT world, environments are growing fast, and if the support for large-scale environments in IoT simulators is limited, it could negatively impact fundamental research efforts, including those targeting large-scale, urban, or even transcontinental IoT frameworks. To address scalability concerns, researchers should focus on enhancing the scalability of simulators, possibly by applying distributed computing techniques or cloud-based platforms, allowing for the simulation of vast IoT networks spanning cities or even continents. Moreover, integrating real-world data streams, especially those emanating from smart city infrastructures, into simulations presents a complex set of challenges. Smart city infrastructures continuously generate vast amounts of data, which not only vary in volume and velocity but also in format, given the diverse sources like sensors, cameras, and traffic systems. A significant challenge is ensuring that simulators can seamlessly process, interpret, and handle this heterogeneous data. Moreover, the real-world data can often be incomplete, thus requiring robust preprocessing and validation mechanisms within the simulators. There are also temporal and spatial intricacies to consider, as data often comes time-stamped and geo-tagged, requiring simulators to manage both dimensions adeptly. Challenges also arise in real-time data integration, efficient storage and retrieval mechanisms, contextual interpretation of data, and managing feedback loops where simulations might influence and alter real-world operations. Addressing these complex challenges is essential for enhancing the realism and applicability of future IoT simulations.

With the development of Vehicular Cloud Computing (VCC), vehicles interact, collaborate, and can leverage their collective computational power more effectively. By transforming vehicles into nodes of a dynamic cloud, VCC harnesses their computational, storage, and sensing capabilities to offer real-time, context-aware services. This significant change, where vehicles consume, produce, and maintain content, challenges the traditional cloud infrastructure’s role. Instead, it consists of a self-organized, adaptive cloud formation that evolves based on the proximity and availability of neighboring vehicles. Given this, an important future trend is beginning to emerge for IoT simulators: the need to simulate vehicular environments accurately. Using IoT simulators along with traffic simulators to simulate Vehicular Ad-hoc Networks (VANET) environments allows smooth integration of mobility, IoT devices, heterogeneity, and battery management in highly heterogeneous and dynamic environments. To the best of our knowledge, there is only one simulator, SimulatorBridger, that explores this. The limited exploration of this intersection between IoT and VANET simulation indicates an urgent need for more in-depth and diverse investigations. Given the significant potential of VANETs and their increasing relevance in modern urban settings, further research is essential. An expanded research scope would both validate the existing study and offer a broader perspective, capturing details potentially not captured by one study. Given the limited research integrating IoT simulators with traffic simulation for VANET environments, there’s a clear necessity for more extensive investigations in this domain.

The integration of real-world data into IoT simulators raises important issues related to data security and privacy. To the best of our knowledge, no simulator supports simulating attacks such as battery drain, as a simulator has to support battery and communication models. There is a lot of research on detecting attacks on log files that are not simulated in real time with an actual attacker using a real communication infrastructure. However, as the Internet of Things environment expands, several security threats are introduced, requiring simulators to simulate these vulnerabilities accurately. The very nature of data from smart city infrastructures and other IoT deployments is often sensitive. This data can encompass personal information about citizens, traffic patterns, energy consumption metrics, and more. Unauthorized access or breach of such data can have significant consequences regarding individual privacy and broader infrastructural operations. To address this challenge, researchers will need to invest more time and effort in developing IoT simulators that are focused on these issues. Simulators should be designed with built-in encryption modules to ensure data security during transit and while in storage. Additionally, as simulators integrate real-world data, data anonymization and sanitization processes should be implemented to prevent the loss of personally identifiable information. Beyond just data protection, simulators should also be equipped to emulate various threat scenarios. Researchers can learn how real-world IoT deployments might respond to security threats by simulating potential attack vectors, vulnerabilities, and corresponding mitigation strategies. This proactive approach, where potential breaches are identified and addressed in a simulated environment, can pave the way for more resilient IoT systems in the real world.

Moreover, while the different applications have distinct feature priorities, it is important to acknowledge that no single simulator identified in the literature comprehensively addresses all these features across all domains. The unique requirements of each domain present a challenge for simulator developers, making it essential for researchers to carefully select or develop simulators tailored to specific use cases within these diverse application areas. Addressing this limitation could pave the way for more versatile and integrated simulation solutions in the future. This limitation in existing simulation capabilities strongly motivates the need for the development of a generic simulation framework Section 8.2.1.

### 8.2. Future Works: Conceptualizing a New Simulator

#### 8.2.1. Generic Simulator by Supporting OSI Layers

The IoT simulators may address aspects related to different OSI layers Figure 1, but each simulator does not cover all layers across all application domains comprehensively. This limitation is due to the vast diversity of requirements, protocols, and technologies across different IoT applications, as shown in Figure 1, which makes it challenging to develop a one-size-fits-all simulator. For instance, smart city IoT simulators like IoTSim-OsmosisRES and SimulatorBridger, in order to accurately simulate traffic and energy management, cover a broad range of the OSI model. While the main focus might be on higher layers: Application, Presentation, Session, and Network due to their direct relevance to application-specific simulations, but the physical layer is not simulated in these simulators. On the other hand, simulators focusing on agriculture may excel in simulating the Application layer’s pest and disease simulation and weather simulation but lack the depth to simulate lower layers like the Data Link or Network layers, which are crucial for detailed network communication simulations required in smart city applications.

However, despite the varying focus on these features within each domain and supporting different OSI layers, it is important to note that no simulator identified in the literature can comprehensively address all these feature requirements across all domains. In other words, no existing IoT simulator can simulate any scenario from any domain in the IoT world. It is possible to move from one domain to another as in SimulatorBridger [4] that moved from the smart home domain to the smart cities domain. But still, it is not able to simulate a scenario in the healthcare domain, which requires adding a new agent to it. This is because there is no existing IoT simulator that is able to simulate all the OSI layers. This gap highlights a significant challenge in the field of simulation, as there is currently no single platform able to simulate any scenario across these diverse IoT applications.

Moreover, the specialized focus of each simulator on certain OSI layers determined by its target application domain reveals a fundamental gap in the field of IoT simulation: the lack of a versatile, multi-layer, cross-domain simulator. Hence, this highlights a significant research opportunity in the field of simulation technology: developing advanced simulators capable of dynamically adapting to simulate any given scenario in any IoT application by supporting and simulating all OSI layers. Researchers and practitioners should be aware of this limitation when selecting simulators for specific use cases and consider the development of more versatile simulators as a valuable area for future work in the field of simulation research.

This can be achieved by considering a simulator that supports reliable IPv6 simulation as [68]. An OMNeT++ simulator is constructed from modules that communicate using message passing. An hierarchical system structure can be created by nesting modules, which makes it possible to construct complex systems from simpler components in a clear and manageable manner. So, the framework is designed to be easily extended with new protocols or devices. The users are able to create their own modules or modify existing ones to meet their specific requirements. Hence, since every block in OMNET++ is simulated, it is possible to combine different modules in order to have a new simulator that is able to simulate different scenarios in IoT applications. There are existing works that combined modules with OMNET++ such as [69,70]. As a result, Omnet++ acts as a platform that schedules events based on specific distributions, and its modular nature makes it possible to advocate creating a simulator that simulates a wide range of Internet of Things scenarios by combining different modules. Achieving this would represent a significant step forward in simulation technology, allowing researchers, developers, and practitioners across the IoT ecosystem greater flexibility and utility.

#### 8.2.2. Secure Simulator by Cybersecurity Enhancements

Based on the current version of SimulatorBridger [4], it is notable that the simulation capabilities are limited, particularly in terms of simulating battery-draining attacks and extending the simulator with new agents, such as attackers and cyber-security diagnostic tools. This simulator cannot support direct communications between IoT devices, as it only facilitates interactions between IoT devices and Cloud nodes via Edge nodes. This architectural limitation is significant given that many current battery-draining attacks involve direct communication between IoT devices in IoT environments. By engaging in overly extensive, unnecessary communication requests or other energy-consuming activities, attackers target another IoT device to drain the battery. To address this, it is necessary to set up a whole IPv6 infrastructure in the simulator, which is currently not available. This gap in functionality means that SimulatorBridger cannot effectively model complex attack scenarios or provide a thorough understanding of security attacks, especially those involving direct communication between IoT devices, which are increasingly common in modern IoT networks. A fundamental understanding of the security of IoT networks can be obtained only by significantly revising such a simulator: due to the simulator’s limited interaction capabilities and the missing IPv6 infrastructure, its applicability is currently constrained. So, the new secure simulator was proposed in [71] that can address this limited interaction capabilities, therefore, support cyber-security detection algorithms.

Due to the importance of simulating cyber threats in different IoT scenarios Section 6, such a simulator should also be able to mimic non-mobility-based IoT nodes. This should be trivial, as this boils down to assuming that such devices will never change their position. In summary, the use case involves deploying edge gateways to detect and mitigate IoT device battery-draining attacks. This is motivated by the need to protect the longevity and functionality of these devices, which are increasingly integral to the operation of modern smart environments.

## 9. Conclusions

It can be concluded that most existing IoT simulators are designed specifically to support specific scenarios with their unique abstractions, indicating a notable gap in having a generic IoT simulator capable of simulating any scenario in different IoT applications. Furthermore, through our systematic analysis, we identify a critical gap in the current IoT simulation frameworks: that is, no simulator supports simulating attacks especially battery depletion attacks, which are growing in importance in IoT systems. As a result of this study, it becomes clear that advanced, versatile platforms for IoT simulation are urgently required. Besides bridging the current gap by supporting simulations across all OSI layers, such platforms should also provide the capability to simulate a wide range of cyber threats, including battery depletion attacks. Addressing these issues will enable more comprehensive and accurate simulations and open a variety of opportunities for researchers to make significant contributions to the field. The future development of IoT simulators is likely to be dynamic and multifaceted, reflecting the complexities and opportunities of the rapidly advancing IoT landscape.

## Figures and Tables

**Table 1:** Existing IoT Simulators.

Tool	Focus	Key Features	Limitations
IoTNetSim	End-to-end IoT services	Detailed modeling of IoT nodes, sensors, and mobility. Supports various protocols. Modular and extendable architecture.	limitations in supporting certain sensor types due to the complexity of modeling their mobility.
EdgeMiningSim	IoT data mining in edge computing	Multi-layered architecture. Supports task offloading and edge server management. High scalability.	Requires substantial computational resources.
Large-scale NB-IoT Simulator	IoT in smart cities	Integrates real geographical data. Tailored for NB-IoT and LTE devices. Discrete-event simulation approach.	Limited to NB-IoT and LTE devices.
ASSIST	Social IoT environments	Models social interactions among IoT devices. Supports common IoT protocols. Scalable for extensive networks.	Primarily supports SIoT environments.
Co-simulator for Smart Grids	Smart grids	Integrates Gridlab-D and CORE. GUI for efficiency and software emulation for fidelity.	Focus on smart grids, limited IoT applicability.
GVSoC	RISC-V-based IoT processors	Event-driven, balances accuracy and speed. Highly configurable for DSE.	Focuses on RISC-V, lacks support for other architectures.
Large-Scale IoT Simulator	IoT systems in urban settings	Simulates thousands of devices. High level of generality.	Limited to application-layer perspective, limits its suitability for testing low-level networking aspects
IoT simulator in [22]	Energy management in city districts	Integrates diverse data sources. Leverages LinkSmart Middleware.	Requires expansion for more IoT devices such as weather and traffic sensors.
LoRa-MAB	Resource allocation in LoRaWAN	Event-driven framework. Provides insights into network performance.	Focus on LoRaWAN, may not cover all IoT scenarios.
Dynamic Co-simulation with Multi-Agent System	Modular IoT system simulation	Enables separate simulation of IoT components. Adaptable and modular.	Complex setup with multiple simulation tools, limitation for adding intelligence to the models
MobIoTSim	Mobile IoT device simulation	Emulates devices, generates real-time data. Connects to cloud gateways.	May not fully replicate real device behavior.
RelIoT	Reliability in IoT networks	Integrates modules for power, performance, and temperature. Estimates device reliability.	Needs support for more complex reliability models.
MoSIoT	IoT healthcare monitoring	MDE for scenario creation. Supports commercial IoT hubs.	Focus on healthcare, may not cover other IoT areas.
Hybrid Simulation-Based in [23]	Large-scale IoT applications	Combines simulation and real-life testing. Utilizes PADS methodology.	Focused on system level, may not address detailed IoT protocols.
SimulateIoT	IoT system design and simulation	DSL for scalable IoT systems. Model-Driven Development.	Limited node mobility and hardware simulation.
SimulateIoT-FIWARE	IoT simulation on FIWARE	Extends SimulateIoT for FIWARE. Generates code for specific FIWARE technology.	Tailored to FIWARE, limited other platform applicability.
MyiFogSim	VM migration in fog computing	Supports VM migration policies. Models mobile users and wireless access points.	Needs improvement in scalability.
EdgeCloudSim	IoT services over Edge and Cloud	Detailed analysis of service time, and energy consumption. Accommodates mobile devices.	Missing nuances of diverse hardware features.
Mercury	Real-time fog computing scenarios	Focuses on low latency, high throughput, and 5G. Data stream analytics and federated computation offloading.	Cloud computing not included in initial approach.
IoTSim-Edge	IoT and edge computing challenges	Models device diversity, protocols, mobility. Supports mobile IoT devices.	Does not consider energy consumption of infrastructure.
SimIoT	Cloud computing and IoT	Models users, data centers, virtual machines. Optimizes message exchanging. Supports heterogeneity.	Lacks real-world IoT implementation and explicit energy efficiency measures.
SimulateIoT-Mobile	IoT environments with mobile nodes	Extends SimulateIoT for mobile scenarios. Utilizes MQTT for mobility management.	Assumes guaranteed connectivity, which may not reflect real-world conditions.
PIoT	Network performance of IoT in cities	Front-end for simulation configuration. Models millions of IoT devices using cellular infrastructure.	Focuses on network performance, less on IoT device energy sources.
Contiki-Cooja	Network simulation for Contiki OS	Enables specification of Contiki motes. Provides crucial network data post-simulation.	Emphasizes hardware and network challenges, not IoT communication models.
ABS-SmartComAgri	Precision agriculture	Manages pesticide usage. Implements smart communication protocols.	Specifically for precision agriculture, not general IoT applications.
FS-IIoTSim	Industrial IoT systems	Supports communication protocols. Scenario modeling and performance evaluation.	Tailored for industrial environments, may not cover broader IoT applications.
IoTSim-Osmosis	Integrated edge-cloud IoT applications	Models dynamic workload transfer. Unified modeling for IoT in edge-cloud environments.	Limited wireless communication layer, fixed IoT device locations.
IoTSim-Osmosis-RES	Sustainable IoT ecosystems	Incorporates renewable energy sources. Models energy management and network infrastructure.	Does not support direct communication between IoT devices.
SimulatorBridger	VANETs in urban mobility	Bridges IoT simulation with traffic simulation. Manages mobility and communications of IoT devices.	Does not support direct communication between IoT devices, lack of supporting security model.

**Table 2:** Classification of IoT Simulators Based on their Operational Domains.

Simulator	Edge Modeling	Fog Modeling	Cloud Modeling
**iFogSim**	✗	✓	✓
**MyiFogSim**	✗	✓	✓
**SimIoT**	✓	✗	✓
**IoTSim-Edge**	✓	✗	✓
**IoTSim-Osmosis**	✓	✗	✓
**IoTSim-OsmosisRES**	✓	✗	✓
**IOTSim**	✓	✗	✓
**CupCarbon**	✓	✗	✗
**MobIoTSim**	✗	✓	✗
**SimulateIoT**	✓	✓	✓
**Contiki-Cooja**	✓	✗	✗

**Table 3:** Comparison of the Features of IoT Simulators Across IoT Applications.

	FS-IIoTSim	IoTSim-Osmosis	IoTSim-OsmosisRES	SimulatorBridger	ABS-SmartComAgri	MoSIoT
**Smart City:**						
Traffic Simulation			✓	✓		
Energy Management		✓	✓	✓		
**Smart Home:**						
User Interaction		✓				
Security Simulation	✓	✓				✓
**Health Care:**						
Patient Monitoring						✓
Electronic Health Records						✓
**IIoT:**						
Fault Detection	✓					
Remote Monitoring and Control	✓					
**Agriculture:**						
Pest and Disease Sim					✓	
Weather Simulation			✓	✓	✓	

**Table 4:** Comparison of IoT Simulators.

Simulation Platform	Language	Open Source	Mobility	SDN	Energy Model	Scalability	RES *
**iFogSim**	Java	✓	✗	✗	✓	✗	✗
**MyiFogSim**	Java	✓	✓	✗	✗	✓	✗
**SimIoT**	Java	✓	✗	✗	✗	✗	✗
**IOTSim**	Java	✓	Limited	✗	✓	✓	✗
**CupCarbon**	Java	✓	Limited	✗	✓	✓	✗
**IoTNetSim**	Java	✓	Limited	✗	✓	✓	✗
**SimulateIoT**	Java	✓	Limited	✗	✓	✗	✗
**EdgeCloudSim**	Java	✓	✓	✗	Limited	✗	✗
**IoTSim-Edge**	Java	✓	✗	✗	✗	✓	✗
**IoTSim-Osmosis**	Java	✓	✗	✓	✓	✓	✗
**NB-IoT Simulator**	Python	✓	✗	✗	✗	✓	✗
**IoTSim-OsmosisRES**	Java	✓	✗	✓	✓	✓	✓
**SimulateIoT-Mobile**	Java	✓	✓	✗	✗	✓	✗
**SimulatorBridger**	Java	✓	✓	✓	✓	✓	✓

* RES: Renewable Energy Sources.

## Data Availability

This article contains no data or material other than the articles used for the review and referenced.
